# Influence of host genetic polymorphisms involved in immune response and their role in the development of Chikungunya disease: a review

**DOI:** 10.1590/1414-431X2023e12557

**Published:** 2023-09-08

**Authors:** W.J.P. Gotay, R.O. Rodrigues, J.N.U. Yaochite

**Affiliations:** 1 Departamento de Análises Clínicas e Toxicológicas, Faculdade de Farmácia, Odontologia e Enfermagem, Universidade Federal do Ceará, Fortaleza, CE, Brasil

**Keywords:** Single nucleotide polymorphisms (SNPs), Chikungunya virus (CHIKV), Chikungunya disease, Immune response

## Abstract

Chikungunya virus (CHIKV) is transmitted by the bite of infected mosquitoes and can cause significant pathogenicity in humans. Moreover, its importance has increased in the Americas since 2013. The primary vectors for viral delivery are the mosquito species *Aedes aegypti* and *Aedes albopictus*. Several factors, including host genetic variations and immune response against CHIKV, influence the outcomes of Chikungunya disease. This work aimed to gather information about different single nucleotide polymorphisms (SNPs) in genes that influence the host immune response during an infection by CHIKV. The viral characteristics, disease epidemiology, clinical manifestations, and immune response against CHIKV are also addressed. The main immune molecules related to this arboviral disease elucidated in this review are *TLR3/7/8*, *DC-SIGN*, *HLA-DRB1/HLA-DQB1, TNF, IL1RN, OAS2/3*, and *CRP.* Advances in knowledge about the genetic basis of the immune response during CHIKV infection are essential for expanding the understanding of disease pathophysiology, providing new genetic markers for prognosis, and identifying molecular targets for the development of new drug treatments.

## Introduction

The chikungunya virus (CHIKV) is a member of the *Alphavirus* genus, belonging to the *Togaviridae* family, whose genetic material is constituted by a single strand of RNA of approximately 12 kb with positive polarity. This virus is the etiologic agent of Chikungunya disease (CHIK) and was first isolated in 1952-1953 during an epidemic in East Africa (Makonde Plateau-Tanzania) (1,2). Between 1960 and 1990, CHIK outbreaks occurred sporadically in Africa and Asia, reappearing more frequently from 2000 onwards and spreading to other countries (3). In 2014, CHIKV was responsible for causing a major epidemic in Brazil, mainly affecting the Northeast region (4). In the Americas alone, about 693,489 suspected cases and 37,480 confirmed cases of CHIK were reported in 2015 (5,6). In 2022, from January until November 23, 2022, a total of 362,021 cases and 77 deaths were reported. The majority of cases have been reported in Brazil (247,537) (7). CHIKV infection may have different clinical presentations. The acute phase of Chikungunya is associated with various symptoms and characterized by the onset of high fever, polyarthritis, and maculopapular rash. The chronic phase is marked by arthritogenic manifestations lasting >3 months and the onset of rheumatic symptoms (8). After the bite of an infected mosquito, CHIKV is inoculated into the blood capillaries and dermis, where it infects resident skin cells such as keratinocytes and dermal fibroblasts, initiating the first cycle of viral replication (9). The main sites of secondary infection are the muscles and joints, causing polyarthralgia/polyarthritis. The incubation period for CHIKV is usually 2 to 7 days in most cases (10). Numerous risk factors for developing severe disease in adult patients have been reported, including age and gender. Moreover, host genetic variation has a significant influence on the immune response and outcome of CHIKV infections. It modulates the disease, influences viral entry into cells, symptom onset and progression, and individual susceptibility or resistance to infection (11,12). Thus, the knowledge of the host profile of genes that influence or contribute to the outcome and susceptibility of CHIKV infection is an essential tool to elucidate the pathophysiology of this arboviral disease and its progression (2,13). Therefore, the present review aimed to identify in the literature the influence of host genetic variations on the immune response during CHIKV infection and the role of genetic polymorphisms on the outcome of the disease.

## General characteristics of Chikungunya virus

CHIKV is an enveloped alphavirus with 60-70 nm in diameter. It belongs to the *Togaviridae* family, and the main vectors in the transmission of CHIKV are female mosquitoes of the *Aedes* genus, more specifically the *Aedes aegypti* and *Aedes albopictus*, which are widely distributed in endemic regions (4,5).

CHIKV has an 11.8 kb-long single-stranded positive-sense RNA (14). The CHIKV genome contains two reading frames (open reading frame, ORF): the 5' ORF, translated from genomic RNA (49S) and coding for four nonstructural proteins (NSP1, NSP2, NSP3, and NSP4), and the 3' ORF, translated from a subgenomic RNA (26S) and coding for a polyprotein later cleaved into the capsid protein, two envelope proteins (E1 and E2), and two small peptides, E3 and 6K (15,16). E1 and E2 are surface glycoproteins, 439 and 423 amino acid-long, respectively (17,18). E1 is responsible for the fusion of the viral membrane and the endosomal membrane, and E2 is involved in receptor binding and subsequent receptor-mediated endocytosis (15,19). The 61-amino acid 6K protein increases cell permeability to monovalent cations and virion budding during infection (15,18). Transframe protein (TF) is produced as a result of the C-terminal extension of 6K protein (20). Nonstructural proteins (NSP1-4) are responsible for viral replication machinery (21).

Four distinct CHIKV genotypes (or lineages) have already been identified and named based on their geographical distribution: i) the West African; ii) the East/Central/South African (ECSA); iii) the Asian, and iv) the Indian Ocean Lineage (IOL) (22,23). Since 2014, the ECSA genotype has been detected in several Brazilian states in the northeastern, southeastern, and northern regions, seriously threatening public health (22).

The female *Aedes* mosquito introduces the virions at the intradermal level through the bite, and they enter the subcutaneous capillaries. There, a local viral replication occurs in susceptible cells such as fibroblasts, endothelial cells, and macrophages (24). Couderc et al. (25) established the development of an animal model and observed that CHIKV replicates first in the liver and target muscles, joints, and skin, resembling the tissue/cell tropism seen on biopsy samples from humans infected with CHIKV. They also observed the spread of the virus to the choroid plexuses and leptomeninges in the central nervous system in severe infections (25).

## Epidemiology of Chikungunya

CHIKV is a zoonotic virus that uses several non-human primates and possibly other vertebrates as amplification hosts (10). Phylogenetic studies of CHIKV suggest that the virus has been circulating enzootically in Africa for centuries or longer (10,26). In addition to evidence of Chikungunya outbreaks in the 18th century, there are descriptions of serial epidemics consistent with the disease in India, dating from the 1820s, 1853, 1871, and 1923, especially in Calcutta but also extending to Burma (10). In 2005, a large outbreak of Chikungunya occurred on islands in the Indian Ocean, reaching several countries in Asia in the following years, causing the infection of more than 1.9 million people (27,28).

CHIKV arrived in the Americas for the first time in 2013. In October of that year, the first autochthonous cases were diagnosed on Saint Martin island in the French Caribbean (29). A major epidemic began that spread to more than 43 American countries, with 1,300,000 cases and 191 deaths in 2014 (4).

In Brazil, the virus was first identified in 2014. As of June 2021, 41,673 probable cases of Chikungunya were notified in Brazil. Of these, 21,127 occurred in the Northeast region, 18,663 in the Southeast region, 605 in the North region, 655 in the South region, and 623 in the Center-West region (30). Globally, as of 23 November 2022, 362,021 cases and 77 deaths were reported. The majority of cases have been reported in Brazil (247,537), India (108,957), Guatemala (1,615), Thailand (842), and Malaysia (688). Deaths have been reported in Brazil (75) and Kenya (1) (7).

## Clinical manifestations of Chikungunya

In most reports, almost all of the infected patients became symptomatic. Acute clinical symptoms usually include high fever (>38.5°C), chills, severe joint and muscle pain, rash, weakness, and headache. Fever is the main symptom in children. Atypical and severe cases were frequently observed in this group of patients, with the disease leading to hyperpigmentation and erythema (31,32). CHIKV can cause persistent infection in macrophages and replicate at low levels for several days, spreading to joint tissues and causing cellular infiltrates, persistent inflammation, and arthritic pain (10). Studies have detected CHIKV RNA in the affected joints, being able to induce arthritis (33).

Studies show that more than 40% of patients progress to the chronic form of the disease. The pathophysiological mechanisms of musculoskeletal pain and chronic arthritis after CHIKV infection are only partially understood (34).

Studies carried out after CHIKV outbreaks in Réunion Island in 2006 and Italy in 2007 showed persistence of myalgia, asthenia, and arthralgia in 60-67% of cases 36 and 12 months after infection, respectively (35). Chronic manifestations may include monoarthritis, oligoarthritis, undifferentiated polyarthritis, and joint involvement mimicking rheumatoid arthritis (36). Fatigue, mood disorders, and sleep disturbances were also common chronic symptoms (37). Long-term arthralgia was typically polyarthralgia (70%), which was generally symmetrical (90%) and highly disabling (77%). These manifestations were often associated with local swelling (63%) or depression (56%) (35).

CHIKV causes an exaggerated inflammatory response and infection of osteoblasts, which resembles rheumatoid arthritis by the similar pattern of leukocyte infiltration, cytokine production, and complement system activation (38). Neurological complications, including encephalitis, optic neuritis, facial paralysis, sensorineural deafness, and Guillain-Barré syndrome, may occur in a variable proportion of patients. Less frequently, CHIKV causes myocarditis, cardiac arrhythmia, severe sepsis, septic shock, and renal failure (39).

## Immune responses to CHIKV

### Innate immune response

The mechanism by which CHIKV initiates the immune response is similar to that observed in other RNA viruses. The innate immune response begins with the recognition of viral RNA by pattern recognition receptors (PRRs) (40,41). In the case of CHIKV, viral RNA activates the Toll-like receptors (TLR-3 and TLR-7) and retinoic acid-inducible gene-like receptors (*RIG-I*) and melanoma differentiation-associated protein 5 (MDA5) (41,42). The signal emitted through the ectodomain of these receptors allows the recruitment of adapter molecules that activate different transcription factors such as nuclear factor (NF)-κB and the interferon regulatory factors (IRFs), inducing the transcription of antiviral interferons (type I IFNs: IFN-α and IFN-β) and proinflammatory cytokines (41,42).

The release of type I IFNs controls viral replication in cells and viral progression during acute infection. Studies show that inefficient type I IFNs signaling is a risk factor for developing severe forms of CHIKV-associated disease (25,33). In humans, IFN-α is released early and is detected on the first day of infection. Moreover, its concentration correlates with plasma viral load (10,43). Experimental models show an early induction of IFN-α, MCP-1/CCL-2, and IL-6, followed by the detection of MIP-1α/CCL-3 and MIP-1β/CCL-4, IFN-γ, and TNF (44,45).

Teo et al. (46) demonstrated the importance of NK cells during acute joint pathology in CHIKV-infected mice and revealed that reduced NK cells activity could underlie the reduced pathology in the host. Different studies showed that in the chronic phase of Chikungunya, the cellular infiltrate in the affected joints is mainly composed of macrophages, NK cells, monocytes, and macrophage-derived osteoclasts (10,47). Osteoclasts and IL-6 may be associated with bone destruction. In most cases, post-CHIKV arthritis is usually non-erosive (35).

Persistent arthralgia was associated with higher levels of IL-6 and granulocyte-macrophage colony-stimulating factor (GM-CSF) (24). A study suggests that γδ T cells play a protective role by limiting the inflammatory response induced by CHIKV, and mice with γδ T cell deficiency showed exacerbated CHIKV infection (48).

Studies show that CHIKV presents different mechanisms to counteract the host's immune response. CHIKV inhibits IFNs signaling through its nonstructural protein nsP2 by blocking the JAK/STAT (signal transducer and activator of transcription) pathway (49,50). Moreover, mutations within nsP2 alter the production of IFN-β, and the nsP2 viral protein can inhibit RNA polymerase II by inducing the degradation of its catalytic subunit RpB1, blocking the expression of cellular genes (51,52).

### Adaptive immune response

Symptoms of acute CHIK are caused by direct cell damage and local inflammation. However, the specific contributions of viral replication and host adaptive immune response to CHIKV infection remain to be fully elucidated (53). The acute phase of the human disease leads to the activation and proliferation of CD8^+^ T cells, whereas the CD4^+^ T cell response is dominant during the chronic phase of Chikungunya (54,55).

T and B cells play an essential role in the immune response against CHIKV and the pathogenesis of CHIKV-associated joint disease. Hawman et al. (56) studied a chronic infection model of Chikungunya using *Rag1^−/−^* mice devoid of mature T and B cells. It was demonstrated that CHIKV RNA persisted in joint-associated tissues. In contrast, CHIKV RNA was cleared from visceral and muscle tissues of wild-type mice, suggesting that an acquired immune system is required for CHIKV clearance (56).

CD4+ T cells were shown to contribute to joint inflammation in the course of CHIKV infection in mice. Carissimo et al. (57) showed that interferon-stimulated Viperin protein expression correlated with viral load. An animal model of CHIKV infection using *Viperin^−/−^* mice has shown that the absence of this protein significantly contributed to promote an increase of the Th1 profile, with increased INFγ secretion in the evaluated tissues, resulting in high-intensity joint inflammation compared to wild-type mice. Regulatory T cells (Tregs) may also be involved in CHIKV infection immunopathogenesis, as Tregs expansion reduces CHIKV disease by selectively inhibiting CHIKV-specific CD4^+^ effector T cells during the chronic phase (58).

In mouse models, CD8^+^ T cell numbers in the joints increase during acute CHIKV infection. However, they do not protect against CHIKV-associated pathologies, as mice deficient in CD8^+^ T cells still develop joint inflammation (57).

The development of anti-CHIKV neutralizing antibodies is essential to control viremia. In humans and mice, the antibody-mediated immune response seems to target the envelope glycoprotein E2 of CHIKV (59,60). Kam et al. (61) found that the early release of IgG3 isotype antibodies *in vivo* dominates the humoral response against CHIKV. The neutralizing effect of IgG3 antibodies was also evident in *in vitro* infection assays. Exposure of CHIKV to patient plasma without IgG3 prevented its inhibitory effect on viral infection of HEK 293T cells. Elderly people have a weaker adaptive response, are more prone to CHIKV infection, and the disease is more severe in these patients (62).

## The role of polymorphisms in genes involved in host immune response during CHIKV infection

**Figure 1:**
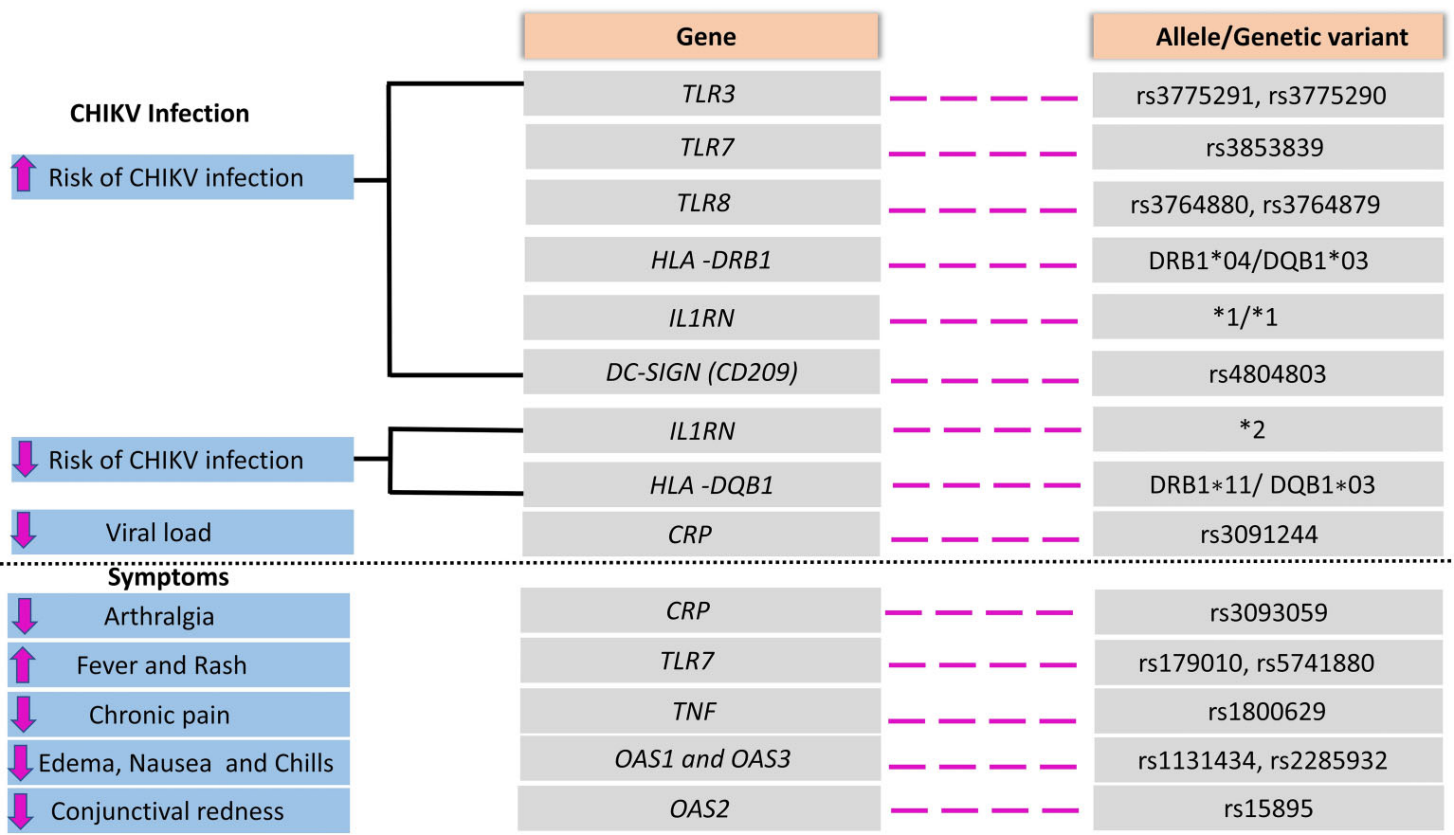
Summary of host genetic polymorphisms involved in the immune response and their association with Chikungunya virus (CHIKV) infection and clinical symptoms of Chikungunya.

Host factors, such as innate and adaptive immune responses, and also genetic background, play an essential role in Chikungunya disease development and progression. Different studies have shown the association of several host genetic polymorphisms that influence the immune response against CHIKV, resulting in susceptibility or resistance to infection. Here, we addressed the main findings about *TLR3/7/8*, *DC-SIGN*, *HLA-DRB1/HLA-DQB1*, *TNF*, *IL-1RN*, *OAS2/3*, and *CRP* genetic polymorphisms in Chikungunya (Figure 1 and Table 1).

**Table 1:** Influence of genetic polymorphisms in genes involved in the immune response against Chikungunya virus (CHIKV) infection and disease outcomes.

Gene	Allele/polymorphism	Chromosome	SNP Type	Association	Reference
*TLR3*	rs3775291 [A/G]	Chr 4	Transition Substitution	AA genotype promotes increased CHIKV infection susceptibility.	(13)
	rs3775290 [T/C]		Transition Substitution	TT genotype increases the risk of arboviruses coinfection. Increases the risk of Chikungunya infection.	(68)
*TLR7*	rs3853839 [C/G]	Chr.X	Transversion Substitution	GC genotype is associated with increased risk of CHIKV infection. Increases the risk of fever and rash.	(11,68)
	rs179010 [T/C]		Transversion Substitution	Increases the risk of fever and rash.	(11)
	rs574880 [G/T]				(11)
*TLR8*	rs3764880 [A/G]	Chr.X	Transversion Substitution	AG genotype is associated with susceptibility to CHIKV infection.	(68)
	rs3764879 [G/C]			Association with increased risk of CHIKV infection.	(11,68)
*HLA II*	DQB1*03: 03/ DRB1*11	Chr.6	Insertion/Deletion (Indels)	Protection against CHIKV infection.	(77,78)
*-DQB1 -DRB1*	DQB1*03DRB1*4			Susceptibility to CHIKV infection.	(78)
*TNF*	rs1800629 [A/G]	Chr.6	Transition Substitution	Associated with decreased persistent joint pain in patients with Chikungunya disease.	(13)
*IL1RN*	*1/*	Chr.2	Tandem Repeat	Association with increased risk of CHIKV infection.	(82)
	*2			Protection against chronic Chikungunya.	(82)
*CD209*	rs4804803 [A /G]	Chr.19	Transition Substitution	Promotes susceptibility to CHIKV infection.	(2,13)
*OAS3*	rs2285932 [C/T]	Chr.12	Transition Substitution	Associated with a reduced risk of chills.	(2)
*OAS1*	rs1131434 [G/G]		Transition Substitution	Associated with reduced risk of edema.	(2)
*OAS2*	rs1732778 [G/A]rs15895 [G/A]		Transition Substitution	Associations with symptoms such as edema, fever, chills, and reduced conjunctival redness.	(2)
*CRP*	rs3091244 [T/C]	Chr.1	Transition Substitution	CT genotype confers protection against CHIKV infection. Patients with CC genotype present higher viral load for CHIKV.	(90)
	rs3093059 [T/C]rs3091244 [T/C]			Genotypes CT and TT are associated with protection against arthralgia produced by CHIKV.	(87)

The polymorphic variants of these genes were initially selected in the following databases and then confirmed by consulting the reported literature. However, to ensure that only reasonably representative studies were used, studies based on a minimum of 100 individuals or more were selected.

### Toll-like receptors (TLR-3, TLR-7, and TLR-8)

Toll-like receptors (TLRs) are proteins that detect invading or endogenous pathogens, emitting signals that initiate the innate and adaptive immune responses (42). They are prototypic pattern recognition receptors (PRRs), which recognize conserved microbial signature molecules known as pathogen-associated molecular patterns (PAMPs) or microbe-associated molecular patterns (MAMPs), which recognize any microbe regardless of their degree of pathogenicity (41,42).

TLRs that recognize bacterial and fungal components are located on the cell surface. In contrast, TLRs that recognize viral or microbial nucleic acids are located in intracellular membranes, such as endosomes or phagosomes (63). Intracellular TLRs (TLR-3, 7, 8, and 9) play crucial roles in response to intracellular pathogens; TLR-3 recognizes double-stranded RNA, TLR-7 and TLR-8 are activated by single-stranded RNA, and TLR-9 senses CpG sequences in DNA molecules (64).

TLR*-*3 is expressed within many cell types, including microglial cells, mast cells, eosinophils, macrophages, NK cells, and dendritic cells, but is less expressed in B and T lymphocytes (65). The *TLR3 L412F* (rs3775291) is considered the main SNP in the LRR domain of the *TLR3* gene and is associated with the alteration of the TLR*-*3 function (66,67).

Bucardo et al. (13) evaluated the role of *TLR3* polymorphisms (rs3775291 A/G) in susceptibility to CHIKV infection and its association with clinical symptoms. Multivariate logistic regression analysis showed that Chikungunya disease cases were significantly more likely to be carriers of the AA genotype than the GG genotype (OR=3.26, 95%CI: 1.15, 9.21), with no association observed for the heterozygous AG genotype.

Sengupta et al. (68) performed a study with co-infected patients (dengue virus and CHIKV) and CHIKV mono-infected patients, and they reported that patients with TT genotype of *TLR3* (rs3775290) exhibited significantly higher susceptibility towards co-infection. The rs3775290 polymorphism of the *TLR3* gene was also evaluated by Dutta et al. (11). However, this genetic variant was not associated with CHIKV susceptibility, cytokines secretion, or clinical symptoms.

TLR7/8 are tandem duplicated genes on the X-chromosome located in the endosome membrane and recognize ssRNA and synthetic oligoribonucleotides (69,70). TLR-7 is highly expressed in plasmacytoid dendritic cells, which are a cell subset that have a plasmacytoid morphology and primarily secrete vast amounts of type I IFNs in response to viral infection (69,70).

The rs3853839 C/G polymorphism of the *TLR7* gene shows a significant difference in GC genotype and its distribution among the group of patients infected with CHIKV compared to the control group (OR=3.44, 95%CI: 2.127-5.57; P<0.00001) (11). In this same study, they also examined the SNP rs3775290 from the *TLR3* gene, and it was associated with the presence of joint pain. The SNPs rs179010, rs5741880, and rs38533839 of the *TLR7* gene were associated with increased fever, joint pain, and rash, respectively. Interestingly, the SNP in the *TLR7* gene associated with fever (rs179010) was also present in patients with CHIKV infection and increased levels of IFN-α. Finally, fever was also associated with an SNP (rs3764880) in the *TLR8* gene (11). The exact function of SNP rs179010 of *TLR7* is not yet fully elucidated, although some studies suggest that this variant may decrease the expression of TLR-7, downregulating the production of autoantibodies or cytokines (71).

Other SNPs in *TLR7* (rs179008 C/A) and *TLR8* (rs3764879 G/C) genes were significantly related to increased susceptibility to CHIKV infection (68). Moreover, in the same study, patients with GC genotypes of *TLR7* (rs3853839) and *TLR8* (rs3764879) and AG genotypes of *TLR8* (rs3764880) were more susceptible to CHIKV mono-infection (68). Other studies have demonstrated that *TLR8* polymorphisms could produce a truncated TLR-8 receptor with a shorter signal peptide, which results in a more rapid decay of its expression or may affect the *in vitro* protein function, impairing NF-κB activation (72).

In conclusion, most of the polymorphisms previously described in the different studies for the *TLR3/7/8* genes increase the risk of Chikungunya infection and may influence susceptibility, disease progression, and clinical outcome among infected people. Those genetic variations or SNPs in the *TLR* genes could induce differential innate immune responses towards the same pathogen.

### DC-SIGN (CD209)

DC-SIGN (dendritic cell-specific intercellular adhesion molecule 3-grabbing nonintegrin) is a type C lectin expressed in macrophages and dendritic cells, with fundamental importance in the immune response due to its possible interaction with viral and bacterial pathogens (2). Like TLRs, DC-SIGN acts as a pattern recognition receptor, promoting phagocytosis in macrophages and dendritic cells (73). This molecule is encoded by the *CD209* gene, and it is located on chromosome 19 and is highly polymorphic (74). Polymorphisms in the *CD209* gene have been associated with increased susceptibility to CHIKV infection. This gene has SNPs that affect the expression of DC-SIGN at position −336 (rs4804803) (2,75).

The rs4804803 variant represents a transition from guanine to adenine at position −336 promoter region of the *CD209* gene, and this SNP can regulate the expression of *DC-SIGN* (76). Bucardo et al. (13) analyzed the polymorphic variant rs4804803 and after a multivariate logistic regression analysis showed that Chikungunya patients were significantly more likely to be carriers of the AG genotype than carriers of the AA wild-type genotype (OR=5.17; 95%CI: 1.6-16.7). The authors also showed that the proportion of individuals carrying the AG genotype of the *DC-SIGN*-encoding gene was higher in the group seropositive for Chikungunya-specific IgG than in the seronegative group (OR=2.79, 95%CI: 0.88-8.9) (13).

Another study conducted by Chaaithanya et al. (2) evaluated the same variant A/G (rs4804803) of the *CD209* gene in patients with Chikungunya and healthy controls. The results showed that the frequency of the GG genotype was significantly higher in CHIKV patients compared to healthy controls (P=0.046). The authors suggested that the rs4804803 G/G genotype might influence the expression of *DC-SIGN* and affect the innate and the development of adaptive immune responses mediated through DCs and hence the possible association with susceptibility to CHIKV infection (2). In summary, the rs4804803 variant of the *DC-SIGN* gene contributes to the disease and the infection by the virus. The results of the studies provide new insight into how host genetics influence susceptibility to Chikungunya infection.

### Human leukocyte antigens

Human leukocyte antigens (HLA) are coded on the short arm of chromosome 6, occupying a large portion of the DNA (∼3,500 kilobases). *HLA* are the most polymorphic genes in the human genome. These polymorphisms allow the immune system to increase the repertoire of peptides presented by HLA molecules, which will affect the susceptibility to infectious diseases (77). There are two main classes of HLA (HLA class I and HLA class II). T cells that express CD8 molecules react with HLA class I molecules on the cell surface of all nucleated cells. HLA class II molecules are usually present only on antigen-presenting cells such as B cells, macrophages, dendritic cells, and Langerhans cells and interact with T CD4^+^ lymphocytes (78). Polymorphism in the *HLA* genes is clustered in the peptide-binding region and affects the repertoire of peptides presented by an HLA molecule to the T cell.

Studies have demonstrated the association of HLA class II alleles with susceptibility or resistance to CHIKV infection. An Indian study by Chaaithanya et al. (77) demonstrated a significantly decreased frequency of the HLA-DQB1*03: 03 allele in CHIKV patients compared with healthy subjects, suggesting these alleles might be associated with reduced risk of developing CHIKV symptoms, or severe disease.

In the two-locus haplotypes analysis by Thanapati et al. (78), the authors showed that DRB1*11/DQB1*03 and DRB1*04/DQB1*03 haplotypes revealed statistically significant associations with resistance and susceptibility to CHIKV infection, respectively.

A prospective follow-up study of 21 Chikungunya patients from Réunion Island suggested the role of CHIKV infection in rheumatoid arthritis initiation. The study showed the prevalence of HLA-DRB1*01 and DRB1*04 alleles in patients who had rheumatoid arthritis after the infection (79). Thus, the studies showed an association of DRB1*11/DQB1*03 and DRB1*04/DQB1*03 as resistant and susceptible haplotypes, respectively, to CHIKV infection. These results suggest that genetic susceptibility and/or resistance to Chikungunya infection may be modulated by HLA class II alleles.

### Tumor necrosis factor

Tumor necrosis factor (TNF), an important proinflammatory cytokine, plays a role in regulating cell differentiation, proliferation, and death, as well as in innate and adaptive immune response. The *TNF* gene is located on chromosome 6 in the class III region of the MHC locus (80). Several SNPs found in the regulatory region influence the transcription of the *TNF* gene and the circulating level of TNF (78).

A study by Bucardo et al. (13) evaluated the variant rs1800629 A/G of the *TNF* gene in patients with Chikungunya. *TNF* genotype distribution was not associated with the risk of clinical Chikungunya disease nor with Chikungunya seropositivity. However, Chikungunya patients with persistent joint pain were significantly less likely to be carriers of the AG genotype than the GG genotype (OR=0.24; 95%CI: 0.074-0.76). The authors suggested that carriers of the AG genotype, expressing higher levels of TNF-α, might be better able to control infection and clear the infection early, leading to lower prevalence of persistent joint pain (13).

Overall, the rs1800629 polymorphism of the *TNF* gene was not associated with susceptibility to Chikungunya disease or seropositivity by the study presented. However, the AG genotype was associated with a higher concentration of TNF-α that helps in the inhibition of viral replication and early control of the infection leading to a decrease in joint pain in individuals with chronic Chikungunya.

### Interleukin-1 receptor antagonist (IL-1RN)

The IL-1 family consists of IL-1α and IL-1β. These cytokines bind the IL-1 receptor (IL-1R) and initiate an inflammation cascade to induce vascular dilation and fever (81). The human interleukin-1 receptor antagonist (*IL1RN*) gene is located on the long arm of chromosome 2, and the IL-1RN molecule is a potent endogenous competitive inhibitor of IL-1α and IL-1β and has an anti-inflammatory role (81). *IL1RN* gene polymorphism results in quantitative differences in both IL-1RN and IL-1β production. The polymorphic region within intron 2 of the IL1RN* gene contains variable numbers of tandem repeats (VNTR) of 86 bp, resulting in five alleles of the IL1RN* (*1-*5), corresponding to 2, 3, 4, 5, and 6 copies of the 86-bp sequence, respectively (81,82).

The study conducted by Tripathy et al. (82) revealed a significant association of the *IL1RN* *1/*1 genotype under a recessive genetic model with the risk of developing CHIKV infection (OR=1.57, 95%CI: 1.06-2.34, P=0.02). Other findings also indicated that IL1RN*2 allele under the dominant model was associated with protection against chronic Chikungunya (OR=0.54, 95%CI: 0.34-0.85, P=0.009). These findings also suggest a role of higher IL-1RN production in the pathogenesis of chronic Chikungunya (82). Ultimately, the results suggest the association of *ILRN VNTR* polymorphism with Chikungunya. Individuals with the IL1RN*1/*1 genotype showed an increased risk of CHIKV infection, whereas the IL1RN*2 allele could have a protective effect against the development of chronic disease.

### Oligoadenylate synthetase (OAS)

Human oligoadenylate synthetase (OAS) is a family of enzymes encoded by three closely linked genes on chromosome 12q24.2 in the following order: small (*OAS1*, p40/p46), medium (*OAS2*, p69/71), and large (*OAS3*, p100) (83,84). *OAS3* efficiently inhibits the growth of CHIKV. The antiviral effect was a blockade in the early stages of virus replication (84). OAS families are formed by homologous enzymes that are encoded by IFN-stimulated genes. The 2'-5'-oligoadenylate synthetase (OAS)/ribonuclease L (RNase L) system is an innate immunity pathway that responds to pathogens and is associated with the induction of viral and cellular RNAs degradation, thereby blocking viral infections (85). Polymorphisms in the genes encoding OAS enzymes are known to influence susceptibility and severity of viral diseases (2,86).

Chaaithanya et al. (2) aimed to investigate the association of *OAS* polymorphisms with susceptibility to CHIKV infection, evaluating samples from 101 CHIKV-infected patients and 101 samples obtained from healthy subjects. The *OAS2* rs1732778 A/A genotype, under a recessive genetic model (A/A *vs* G/G+G/A), was significantly associated with a reduced risk of nausea [OR with 95%CI: 0.16 (0.05-0.57), P-value=0.0028] (2). The *OAS2* rs15895 G/A genotype and *OAS3* rs2285932 C/T genotype in an overdominant model were significantly associated with a reduced risk of chills. The *OAS3* rs2285932 T allele in a dominant model (C/T+T/T *vs* C/C) and *OAS1* rs1131434 G/G genotype under a recessive genetic model were associated with a reduced risk of edema. *OAS2* rs15895 G/A in an overdominant model was associated with a reduced risk of conjunctival redness. Thus, the authors suggested that *OAS* gene cluster polymorphisms were associated with a reduced risk of developing nausea, chills, edema, and conjunctival redness in CHIKV infection (2).

Finally, this study suggested that SNPs in the *OAS* gene cluster are associated with the risk of developing clinical symptoms in patients infected with CHIKV. Further follow-up studies are needed to evaluate the role of these genes in association with clinical symptoms in CHIKV patients.

### C-reactive protein

C-reactive protein (CRP), an acute phase protein, is genetically located in the region of chromosome 1q23.2 and has been previously used as an inflammatory and infectious biomarker in clinical contexts (87). The main stimulus factor for the liver production of CRP is the IL-6 cytokine. Moreover, TNF and IL-1β, among others, act synergistically with IL-6, exacerbating the stimulus for CRP production (88,89).

Sengupta et al. (90) investigated the role of polymorphisms in the gene encoding CRP and whether these SNPs influence the level of serum CRP concentration in patients with mono-infection (dengue virus infection/n=201 or CHIKV infection/n=167) and patients co-infected with both viruses (n=128). Patients with CT genotype of rs3093059 and with TT genotype of rs3091244 were more susceptible to co-infection (OR=1.951/P=0.0378 and OR=4.381/P=0.0138). Patients with the CT genotype of the rs3091244 variant exhibited protection against CHIKV infection (OR=0.3611; P=0.278) and increased CRP levels compared to those with the CC genotype. Moreover, the same study showed that individuals with the CC genotype (rs3091244) presented a higher viral load for CHIKV (OR=2.643; P=0.0313). Thus, CRP genotype might play a significant role in determining serum-CRP concentration, viral load, and dengue-CHIKV mono/co-infection (90).

The same research group published a recent article showing the influence of *CRP* rs3093059 and rs3091244 polymorphisms in the prognosis of post-Chikungunya chronic arthritis (87). The study was conducted with 167 Chikungunya patients and 102 healthy subjects. Statistical analysis revealed that individuals with the TC genotype of rs3091244 were less susceptible to CHIKV infection (P=0.0126). Moreover, CT (rs3093059) and TT (rs3091244) genotypes were significantly associated with patients without any arthralgic manifestations (P<0.0001, P=0.0004, respectively) (87).

Thus, the findings shown in these studies suggested that both serum biomarker levels and genetic factors played an essential role in the development of post-Chikungunya chronic arthritis among infected patients. These markers could be used to assess patients and identify their potential to develop chronic arthritis.

## Conclusion

This review presented several polymorphisms in genes responsible for encoding transmembrane proteins, enzymes, cell mediators, recognition molecules, and cytokines essential to host immune response against CHIKV infection. SNPs in these genes can alter an individual's gene profile and influence the pathogenesis and progression of Chikungunya disease. These polymorphic variants can increase the risk of infection in the host or even decrease the risk of infection, having a protective effect. The analysis of genetic variations in different genes that influence the immune response can serve as a valuable strategy to identify patients at increased risk of infection by a specific arbovirus, which may lead to more aggressive treatment to prevent the development of severe clinical manifestations.

## References

[1] Naresh Kumar CVM, Sai Gopal DVR (2010). Reemergence of Chikungunya virus in Indian subcontinent. Indian J Virol.

[2] Chaaithanya IK, Muruganandam N, Surya P, Anwesh M, Alagarasu K, Vijayachari P (2016). Association of oligoadenylate synthetase gene cluster and DC-SIGN (CD209) gene polymorphisms with clinical symptoms in Chikungunya virus infection. DNA Cell Biol.

[3] Burt FJ, Chen W, Miner JJ, Lenschow DJ, Merits A, Schnettler E (2017). Chikungunya virus: an update on the biology and pathogenesis of this emerging pathogen. Lancet Infect Dis.

[4] Weaver SC, Lecuit M (2015). Chikungunya virus and the global spread of a mosquito-borne disease. N Engl J Med.

[5] Giménez-Richarte Á, de Salazar MO, Arbona C, Giménez-Richarte MP, Collado M, Fernández PL (2022). Prevalence of Chikungunya, Dengue and Zika viruses in blood donors: a systematic literature review and meta-analysis. Blood Transfus.

[6] Ferreira JM, Santos LDS, Oliveira SP, Dos Santos BRC, Dos Santos ACM, de Moura EL (2021). Chikungunya virus infection outcome: a systematic review of host genetics. Immunol Invest.

[7] ECDC (European Centre for Disease Prevention and Control) Chikungunya worldwide overview. https://www.ecdc.europa.eu/en/chikungunya-monthly.

[8] Gasque P, Bandjee MCJ, Reyes MM, Viasus D (2016). Chikungunya pathogenesis: from the clinics to the bench. J Infect Dis.

[9] Teng TS, Kam YW, Lee B, Hapuarachchi HC, Wimal A, Ng LC (2015). A systematic meta-analysis of immune signatures in patients with acute Chikungunya virus infection. J Infect Dis.

[10] Weaver SC, Osorio JE, Livengood JA, Chen R, Stinchcomb DT (2012). Chikungunya virus and prospects for a vaccine. Expert Rev Vaccines.

[11] Dutta SK, Tripathi A (2017). Association of toll-like receptor polymorphisms with susceptibility to Chikungunya virus infection. Virology.

[12] Mickienė A, Pakalnienė J, Nordgren J, Carlsson B, Hagbom M, Svensson L (2014). Polymorphisms in chemokine receptor 5 and Toll-like receptor 3 genes are risk factors for clinical tick-borne encephalitis in the Lithuanian population. PLoS One.

[13] Bucardo F, Reyes Y, Morales M, Briceão R, González F, Lundkvist Å (2021). Association of genetic polymorphisms in DC-SIGN, toll-like receptor 3, and tumor necrosis factor α genes and the Lewis-negative phenotype with Chikungunya infection and disease in Nicaragua. J Infect Dis.

[14] Metz SW, Geertsema C, Martina BE, Andrade P, Heldens JG, van Oers MM (2011). Functional processing and secretion of Chikungunya virus E1 and E2 glycoproteins in insect cells. Virol J.

[15] Jose J, Snyder JE, Kuhn RJ (2009). A structural and functional perspective of alphavirus replication and assembly. Future Microbiol.

[16] Thiberville SD, Moyen N, Dupuis-Maguiraga L, Nougairede A, Gould EA, Roques P (2013). Chikungunya fever: epidemiology, clinical syndrome, pathogenesis and therapy. Antiviral Res.

[17] Khan AH, Morita K, Parquet MDC, Hasebe F, Mathenge EGM, Igarashi A (2002). Complete nucleotide sequence of Chikungunya virus and evidence for an internal polyadenylation site. J Gen Virol.

[18] Voss JE, Vaney MC, Duquerroy S, Vonrhein C, Girard-Blanc C, Crublet E (2010). Glycoprotein organization of Chikungunya virus particles revealed by X-ray crystallography. Nature.

[19] Carvalho CAM, Silva JL, Oliveira AC, Gomes AMO (2017). On the entry of an emerging arbovirus into host cells: Mayaro virus takes the highway to the cytoplasm through fusion with early endosomes and caveolae-derived vesicles. PeerJ.

[20] Ramsey J, Mukhopadhyay S (2017). Disentangling the frames, the state of research on the alphavirus 6K and TF proteins. Viruses.

[21] Tomar S, Hardy RW, Smith JL, Kuhn RJ (2006). Catalytic core of alphavirus nonstructural protein nsP4 possesses terminal adenylyltransferase activity. J Virol.

[22] Fabri AA, Rodrigues CDS, dos Santos CC, Chalhoub FLL, Sampaio SA, Faria NRC (2020). Co-circulation of two independent clades and persistence of chikv-ecsa genotype during epidemic waves in Rio de Janeiro, Southeast Brazil. Pathogens.

[23] Agarwal A, Dash PK, Singh AK, Sharma S, Gopalan N, Rao PVL (2014). Evidence of experimental vertical transmission of emerging novel ECSA genotype of Chikungunya virus in Aedes aegypti. PLoS Negl Trop Dis.

[24] Chow A, Her Z, Ong EKS, Chen J, Dimatatac F, Kwek DJC (2011). Persistent arthralgia induced by Chikungunya virus infection is associated with interleukin-6 and granulocyte macrophage colony-stimulating factor. J Infect Dis.

[25] Couderc T, Chrétien F, Schilte C, Disson O, Brigitte M, Guivel-Benhassine F (2008). A mouse model for Chikungunya: young age and inefficient type-I interferon signaling are risk factors for severe disease. PLoS Pathog.

[26] Tsetsarkin KA, Chen R, Weaver SC (2016). Interspecies transmission and Chikungunya virus emergence. Curr Opin Virol.

[27] Khongwichit S, Chansaenroj J, Chirathaworn C, Poovorawan Y (2021). Chikungunya virus infection: molecular biology, clinical characteristics, and epidemiology in Asian countries. J Biomed Sci.

[28] Gratz NG (2004). Critical review of the vector status of *Aedes albopictus*. Med Vet Entomol.

[29] Leparc-Goffart I, Nougairede A, Cassadou S, Prat C, De Lamballerie X (2014). Chikungunya in the Americas. Lancet.

[30] Brasil (2021). Monitoramento dos casos de arboviroses urbanas causados por vírus transmitidos pelo mosquito Aedes (Dengue, Chikungunya E Zika), semanas epidemiológicas 1 a 42, 2021. Bol Epidemiológico Arboviroses.

[31] Contopoulos-Ioannidis D, Newman-Lindsay S, Chow C, LaBeaud AD (2018). Mother-to-child transmission of Chikungunya virus: A systematic review and meta-analysis. PLoS Negl Trop Dis.

[32] Torres JR, Falleiros-Arlant LH, Dueãas L, Pleitez-Navarrete J, Salgado DM, Brea-Del Castillo J (2016). Congenital and perinatal complications of Chikungunya fever: a Latin American experience. Int J Infect Dis.

[33] Narendra SC, Chalise JP, Höök N, Magnusson M (2014). Dendritic cells activated by double‐stranded RNA induce arthritis via autocrine type I IFN signaling. J Leukoc Biol.

[34] Javelle E, Ribera A, Degasne I, Gaüzàre BA, Marimoutou C, Simon F (2015). Specific management of post-chikungunya rheumatic disorders: a retrospective study of 159 cases in Reunion Island from 2006-2012. PLoS Negl Trop Dis.

[35] Noret M, Herrero L, Rulli N, Rolph M, Smith PN, Li RW (2012). Interleukin 6, RANKL, and osteoprotegerin expression by Chikungunya virus-infected human osteoblasts. J Infect Dis.

[36] Madariaga M, Ticona E, Resurrecion C (2016). Chikungunya: bending over the Americas and the rest of the world. Braz J Infect Dis.

[37] Paixão ES, Rodrigues LC, Costa MCN, Itaparica M, Barreto F, Gérardin P (2018). Chikungunya chronic disease: a systematic review and meta-analysis. Trans R Soc Trop Med Hyg.

[38] Burt F, Chen W, Mahalingam S (2014). Chikungunya virus and arthritic disease. Lancet Infect Dis.

[39] Amaral JK, Taylor PC, Teixeira MM, Morrison TET, Schoen RT (2019). The clinical features, pathogenesis and methotrexate therapy of chronic Chikungunya arthritis. Viruses.

[40] Thompson CD, Matta B, Barnes BJ (2018). Therapeutic targeting of IRFs: pathway-dependence or structure-based?. Front Immunol.

[41] He X, Jia H, Jing Z, Liu D (2013). Recognition of pathogen-associated nucleic acids by endosomal nucleic acid-sensing toll-like receptors. Acta Biochim Biophys Sin (Shangai).

[42] Blasius AL, Beutler B (2010). Intracellular toll-like receptors. Immunity.

[43] Schilte C, Couderc T, Chretien F, Sourisseau M, Gangneux N, Guivel-Benhassine F (2010). Type I IFN controls chikungunya virus via its action on nonhematopoietic cells. J Exp Med.

[44] Labadie K, Larcher T, Joubert C, Mannioui A, Delache B, Brochard P (2010). Chikungunya disease in nonhuman primates involves long-term viral persistence in macrophages. J Clin Invest.

[45] Poo YS, Nakaya H, Gardner J, Larcher T, Schroder WA, Le TT (2014). CCR2 deficiency promotes exacerbated chronic erosive neutrophil-dominated Chikungunya virus arthritis. J Virol.

[46] Teo TH, Her Z, Tan JJL, Lum FM, Lee WWL, Chan YH (2015). Caribbean and La Réunion Chikungunya virus isolates differ in their capacity to induce proinflammatory Th1 and NK cell responses and acute joint pathology. J Virol.

[47] Dupuis-Maguiraga L, Noret M, Brun S, Le Grand R, Gras G, Roques P (2012). Chikungunya disease: infection-associated markers from the acute to the chronic phase of arbovirus-induced arthralgia. PLoS Negl Trop Dis.

[48] Long KM, Ferris MT, Whitmore AC, Montgomery SA, Thurlow LR, McGee CE (2016). γδ T cells play a protective role in Chikungunya virus-induced disease. J Virol.

[49] Lucas-Hourani M, Lupan A, Despràs P, Thoret S, Pamlard O, Dubois J (2013). A phenotypic assay to identify Chikungunya virus inhibitors targeting the nonstructural protein nsP2. J Biomol Screen.

[50] Fros JJ, Liu WJ, Prow NA, Geertsema C, Ligtenberg M, Vanlandingham DL (2010). Chikungunya virus nonstructural protein 2 inhibits type I/II interferon-stimulated JAK-STAT signaling. J Virol.

[51] Meshram CD, Lukash T, Phillips AT, Akhrymuk I, Frolova EI, Frolov I (2019). Lack of nsP2-specific nuclear functions attenuates Chikungunya virus replication both *in vitro* and *in vivo*. Virology.

[52] Akhrymuk I, Kulemzin S V, Frolova EI (2012). Evasion of the innate immune response: the Old World alphavirus nsP2 protein induces rapid degradation of Rpb1, a catalytic subunit of RNA polymerase II. J Virol.

[53] Dhanwani R, Khan M, Alam SI, Rao PVL, Parida M (2011). Differential proteome analysis of Chikungunya virus‐infected new‐born mice tissues reveal implication of stress, inflammatory and apoptotic pathways in disease pathogenesis. Proteomics.

[54] Wauquier N, Becquart P, Nkoghe D, Padilla C, Ndjoyi-Mbiguino A, Leroy EM (2011). The acute phase of Chikungunya virus infection in humans is associated with strong innate immunity and T CD8 cell activation. J Infect Dis.

[55] Poh CM, Chan YH, Ng LFP (2020). Role of T cells in Chikungunya virus infection and utilizing their potential in anti-viral immunity. Front Immunol.

[56] Hawman DW, Stoermer KA, Montgomery SA, Pal P, Oko L, Diamond MS (2013). Chronic joint disease caused by persistent Chikungunya virus infection is controlled by the adaptive immune response. J Virol.

[57] Carissimo G, Teo TH, Chan YH, Lee CYP, Lee B, Torres-Ruesta A (2019). Viperin controls Chikungunya virus-specific pathogenic T cell IFNγ Th1 stimulation in mice. Life Sci Alliance.

[58] Lee WWL, Teo TH, Her Z, Lum FM, Kam YW, Haase D (2015). Expanding regulatory T cells alleviates Chikungunya virus-induced pathology in mice. J Virol.

[59] Chua CL, Sam IC, Chiam CW, Chan YF (2017). The neutralizing role of IgM during early Chikungunya virus infection. PLoS One.

[60] Weber C, Büchner SM, Schnierle BS (2015). A small antigenic determinant of the Chikungunya virus E2 protein is sufficient to induce neutralizing antibodies which are partially protective in mice. PLoS Negl Trop Dis.

[61] Kam YW, Simarmata D, Chow A, Her Z, Teng TS, Ong EKS (2012). Early appearance of neutralizing immunoglobulin G3 antibodies is associated with Chikungunya virus clearance and long-term clinical protection. J Infect Dis.

[62] Amdekar S, Parashar D, Alagarasu K (2017). Chikungunya virus-induced arthritis: role of host and viral factors in the pathogenesis. Viral Immunol.

[63] Hennessy EJ, Parker AE, O'neill LAJ (2010). Targeting Toll-like receptors: emerging therapeutics?. Nat Rev Drug Discov.

[64] Diebold SS, Brencicova E (2013). Nucleic acids and endosomal pattern recognition: how to tell friend from foe?. Front Cell Infect Microbiol.

[65] Dunlevy F, McElvaney NG, Greene CM (2010). TLR3 sensing of viral infection. Open Infect Dis J.

[66] Ranjith-Kumar CT, Miller W, Sun J, Xiong J, Santos J, Yarbrough I (2007). Effects of single nucleotide polymorphisms on Toll-like receptor 3 activity and expression in cultured cells. J Biol Chem.

[67] Blasius AL, Beutler B (2010). Intracellular toll-like receptors. Immunity.

[68] Sengupta S, Mukherjee S, Bhattacharya N, Tripathi A (2021). Differential genotypic signatures of Toll-like receptor polymorphisms among dengue-chikungunya mono-and co-infected Eastern Indian patients. Eur J Clin Microbiol Infect Dis.

[69] Khanmohammadi S, Rezaei N (2021). Role of Toll‐like receptors in the pathogenesis of COVID‐19. J Med Virol.

[70] Rauseo DM, Fernández-Mestre M (2019). X-linked Toll-like receptor 7 polymorphism associated with susceptibility to Chikungunya Fever. Asian Pac J Trop Med.

[71] Xiao W, Liu Z, Lin J, Li J, Wu K, Ma Y (2015). Association of Toll‐like receptor 7 and 8 gene polymorphisms with Graves' disease in Chinese Cantonese population. Tissue Antigens.

[72] Gantier MP, Irving AT, Kaparakis‐Liaskos M, Xu D, Evans VA, Cameron PU (2010). Genetic modulation of TLR8 response following bacterial phagocytosis. Hum Mutat.

[73] Ojeda N, Salazar C, Cárdenas C, Marshall SH (2020). Expression of DC-SIGN-like C-Type lectin receptors in Salmo salar. Dev Comp Immunol.

[74] Fang X, Hu Z, Shang W, Zhu J, Xu C, Rao X (2012). Genetic polymorphisms of molecules involved in host immune response to dengue virus infection. FEMS Immunol Med Microbiol.

[75] Alagarasu K, Damle IM, Bachal R V, Mulay AP, Shah PS, Dayaraj C (2013). Association of promoter region polymorphisms of CD209 gene with clinical outcomes of dengue virus infection in Western India. Infect Genet Evol.

[76] Pabalan N, Chaisri S, Tabunhan S, Phumyen A, Jarjanazi H, Steiner TS (2018). Associations of DC-SIGN (CD209) promoter-336G/A polymorphism (rs4804803) with dengue infection: A systematic review and meta-analysis. Acta Trop.

[77] Chaaithanya IK, Muruganandam N, Anwesh M, Rajesh R, Ghosal SR, Kartick C (2013). HLA class II allele polymorphism in an outbreak of Chikungunya fever in middle Andaman, India. Immunology.

[78] Thanapati S, Hande A, Das R, Gurav Y, Tripathy AS (2014). Association of human leukocyte antigen class II allele and haplotypes in Chikungunya viral infection in a western Indian population. Trans R Soc Trop Med Hyg.

[79] Bouquillard E, Combe B (2009). A report of 21 cases of rheumatoid arthritis following Chikungunya fever. A mean follow-up of two years. Joint Bone Spine.

[80] Qidwai T, Khan F (2011). Tumour necrosis factor gene polymorphism and disease prevalence. Scand J Immunol.

[81] Nicklin MJH, Weith A, Duff GW (1994). A physical map of the region encompassing the human interleukin-1α, interleukin-1β, and interleukin-1 receptor antagonist genes. Genomics.

[82] Tripathy AS, Ganu MA, Sonam L, Alagarasu K, Walimbe AM, Thanapati S (2019). Association of IL1RN VNTR polymorphism with Chikungunya infection: a study from Western India. J Med Virol.

[83] Hovanessian AG, Justesen J (2007). The human 2′-5′ oligoadenylate synthetase family: unique interferon-inducible enzymes catalyzing 2′-5′ instead of 3′-5′ phosphodiester bond formation. Biochimie.

[84] Bréhin AC, Casadémont I, Frenkiel MP, Julier C, Sakuntabhai A, Despràs P (2009). The large form of human 2′, 5′-Oligoadenylate Synthetase (OAS3) exerts antiviral effect against Chikungunya virus. Virology.

[85] Kristiansen H, Gad HH, Eskildsen-Larsen S, Despres P, Hartmann R (2011). The oligoadenylate synthetase family: an ancient protein family with multiple antiviral activities. J Interferon Cytokine Res.

[86] Sadler AJ, Williams BRG (2008). Interferon-inducible antiviral effectors. Nat Rev Immunol.

[87] Sengupta S, Bhattacharya N, Tripathi A (2022). Increased CRP, anti-CCP antibody, IL-2R, COMP levels in prognosis of post-chikungunya chronic arthritis and protective role of their specific genotypes against arthritic manifestation. Virus Res.

[88] Volanakis JE (2001). Human C-reactive protein: expression, structure, and function. Mol Immunol.

[89] Verma S, Li SH, Badiwala MV, Weisel RD, Fedak PWM, Li RK (2002). Endothelin antagonism and interleukin-6 inhibition attenuate the proatherogenic effects of C-reactive protein. Circulation.

[90] Sengupta S, Bhattacharya N, Tripathi A (2022). Association of C-reactive protein polymorphisms with serum-CRP concentration and viral load among dengue-chikungunya mono/co-infected patients. Antiviral Res.

